# The landscape of d16HER2 splice variant expression across HER2-positive cancers

**DOI:** 10.1038/s41598-019-40310-5

**Published:** 2019-03-05

**Authors:** Chiara Costanza Volpi, Filippo Pietrantonio, Annunziata Gloghini, Giovanni Fucà, Silvia Giordano, Simona Corso, Giancarlo Pruneri, Maria Antista, Chiara Cremolini, Elena Fasano, Serena Saggio, Simona Faraci, Maria Di Bartolomeo, Filippo de Braud, Massimo Di Nicola, Elda Tagliabue, Serenella Maria Pupa, Lorenzo Castagnoli

**Affiliations:** 1 Department of the Pathology and Laboratory Medicine, Fondazione IRCCS Istituto Nazionale dei Tumori, Milan, Italy; 2 Department of Oncology and Hemato-oncology, Fondazione IRCCS Istituto Nazionale dei Tumori, Milan, Italy; 3 Faculty of Medicine, Oncology and Hemato-Oncology Department, Università degli Studi di Milano, Milan, Italy; 4 Department of Oncology, University of Torino and Candiolo Cancer Institute, FPO-IRCC, Candiolo, Italy; 5 Unit of Medical Oncology, Azienda Ospedaliera-Universitaria Pisana, Pisa, Italy; 6 Molecular Targeting Unit, Department of Research, Fondazione IRCCS Istituto Nazionale dei Tumori, Milan, Italy

## Abstract

The HER2 splice variant characterized by the deletion of exon 16 and denominated as d16HER2, is associated with HER2-positive breast cancer (BC) aggressiveness, stemness, and trastuzumab susceptibility and is considered to be a “flag” of HER2 dependence. However, with the exception of quantitative real-time PCR analysis, easily reproducible assays are still lacking to clinically detect and quantify the d16HER2 expression. Further, no data on d16HER2 expression and its potential role are available in HER2-positive gastrointestinal malignancies. Here, we used a novel RNA *in situ* hybridization technique (BaseScope) to discriminate d16HER2 variant expression from the wild type isoform (WTHER2) and to assess their levels across different HER2-positive histological samples. Our results demonstrate the existence of outliers, with d16HER2 mRNA high scores restricted to HER2-positive gastric cancer (GC) and colorectal cancer (CRC) coupled with increased d16HER2 expression compared with BC. Consistent with previously reported data on BC, experiments performed in HER2-positive GC patient-derived xenografts suggest that increased d16HER2 expression is associated with a clinical benefit/response to single-agent trastuzumab. Therefore, d16HER2 may be considered as a “flag” of HER2 dependence in GC and can be clinically investigated as a marker of trastuzumab susceptibility in several other HER2-driven cancers, including CRC. As a clinical proof-of-concept, we indicate that high d16HER2 mRNA scores are exclusively found in patients with a long-term benefit from trastuzumab exceeding 12 months (clinical “outliers”), and that d16HER2 expression is also increased in circulating tumor-released exosomes obtained from baseline plasma samples of long-term responders.

## Introduction

HER2 overexpression/amplification is a therapeutic target for trastuzumab-based treatment in breast, gastro-esophageal and colorectal cancers^1–3^. Tumor biology, prognosis and treatment response display relevant differences when comparing HER2-positive breast cancer (BC) with gastrointestinal cancers^4,5^. On one hand, novel HER2 dual blockade strategies based on the combination of trastuzumab plus pertuzumab (humanized monoclonal antibodies) have entered the treatment landscape of BC both in metastatic, adjuvant and neoadjuvant settings^6–8^, and additional blockbuster drugs are under clinical development^9,10^. On the other hand, HER2 is often heterogeneous and associated with other co-existing drivers in gastric cancer (GC), therefore limiting long-term benefit from trastuzumab in most patients with metastatic disease^4,11^. In contrast, dual HER2 blockade has demonstrated promising activity exclusively in KRAS wild-type metastatic colorectal cancers (CRC)^3,12^. Indeed, primary or acquired resistance to trastuzumab-based therapies is a relevant challenge in clinical practice^4,5,11,13^. Active research is needed to validate and potentially target resistance mechanisms, including intra-tumor occurrence of distinct truncated/genetically altered members of the HER2-derived proteome. Co-expression of full-length/wild-type HER2 (WTHER2) with carboxy-terminal truncated fragments^14^, activating mutations^15^ or splice variants^16^ contributes to the heterogeneity of HER2-driven cancers^17^. In particular, the d16HER2 splice variant, which is expressed as a proportion (approximately 2–9%) of WTHER2 in human HER2-positive BC^18,19^, may have a crucial pathobiological role given that the post-transcriptional loss of 48 nucleotides in the extracellular domain induces the formation of stable and constitutively active HER2 homodimers on the tumor cell surface^18,20^. d16HER2 influences not only tumor aggressiveness and trastuzumab susceptibility^19,21^ but also stem properties^22^ and the metabolic profile^23^ of HER2-positive BC cells. Indeed, d16HER2 expression/activation strongly reflects a status of HER2 oncogenic signaling dependence (HER2 addiction)^19,23–25^, representing a novel and potentially clinically useful biomarker. However, easily reproducible assays are still lacking to clinically detect and quantify d16HER2 expression, and no data on d16HER2 expression and its potential role are available in HER2-positive gastrointestinal malignancies.

Given the inability to specifically detect and discriminate the d16HER2 splice variant from the WTHER2 isoform at the protein level in formalin-fixed paraffin embedded (FFPE) samples and awareness of the poor clinical applicability of quantitative real-time PCR (qPCR) in FFPE tissues^18–20,22,24–26^, we tested a tissue-based method to detect and measure d16HER2 and WTHER2 expression in the clinical setting. Specifically, we established a novel mRNA bright-field *in situ* hybridization (ISH) technique that detects short RNA targets and exon junctions for the analysis of splice variants (BaseScope). In this context, unlike grind-and-bind RNA analysis methods such as qPCR, this ISH technique brings the benefits of *in situ* analysis to RNA biomarkers and may enable rapid development of RNA ISH-based molecular diagnostic assays^27^. Among all available RNA based morphological assays, BaseScope offers the unique opportunity of scoring and potentially comparing d16HER2 expression in FFPE samples. Further, we also tested liquid biopsies as a possible dynamic and non-invasive tool to assess d16HER2 and WTHER2 transcript expression in tumor-released circulating exosomes. The tumor-derived exosomes, representing one major component of the liquid biopsy analysis, are ubiquitously released in body fluids of cancer patients, and act as an “alternative tool” that allows for molecular and genetic profiling of parent tumor cells and are promising clinically relevant surrogates of cancer cells^28,29^.

In this pilot study, we investigated the landscape of d16HER2 expression across different HER2-positive cancers with direct comparison between BC and gastrointestinal malignancies and parallel assessments at both tissue and liquid biopsy levels.

## Results

### d16HER2 expression in pre-clinical models

The specificity of the ISH probes was initially proven in MCF7-Mock BC cells, which express constitutively low levels of WTHER2 and lack d16HER2, transduced with lentiviral vectors encoding for the empty vector or with the human d16HER2 (MCF7-d16) or WTHER2 (MCF7-WT) genes^22^. Three different HER2 mRNA probes were used in the study to specifically discriminate WTHER2 from the d16HER2 isoform. The d16HER2 probe recognizes the exon junction between exons 15 and 17 targeting the d16HER2 splice variant mRNA; the WTHER2 probe recognizes the exon junction between exons 15 and 16 targeting WTHER2 mRNA; the common region (CR) HER2 probe recognizes the exon junction between exons 17 and 18, targeting a region shared in d16HER2 and WTHER2 mRNA (Supplementary Fig. 1A–D). Consistent with data obtained from qPCR analyses (Supplementary Fig. 2A), immunohystochemistry (IHC) (Supplementary Fig. 2B,F,J; Supplementary Table S1) and mRNA ISH (Supplementary Fig. 2C–E; Supplementary Table S1) bioassays revealed that MCF7-Mock cells exhibited low (score 1) or basal (score 0) signals for both WTHER2 and d16HER2 transcripts. On the contrary, cells transduced with WTHER2 exhibited a score 4 for both CRHER2 and WTHER2 probes (Supplementary Fig. 2G,H; Supplementary Table S1), whereas these cells were negative (score 0) for the d16HER2 probe (Supplementary Fig. 2I; Supplementary Table S1). Such findings were confirmed by IHC (Supplementary Fig. 2F; Supplementary Table S1). On the contrary, MCF7-d16-infected cells provided evidence for a score 4 for the CRHER2 and d16HER2 probes (Supplementary Fig. 2K,M; Supplementary Table S1) as further validated by IHC (Supplementary Fig. 2J). However, these cells exhibited score 1 for the WTHER2 probe (Supplementary Fig. 2L; Supplementary Table S1). Overall, these data sustained the specificity of the tested probes in identifying the d16 and WT forms of HER2.

**Figure 1:**
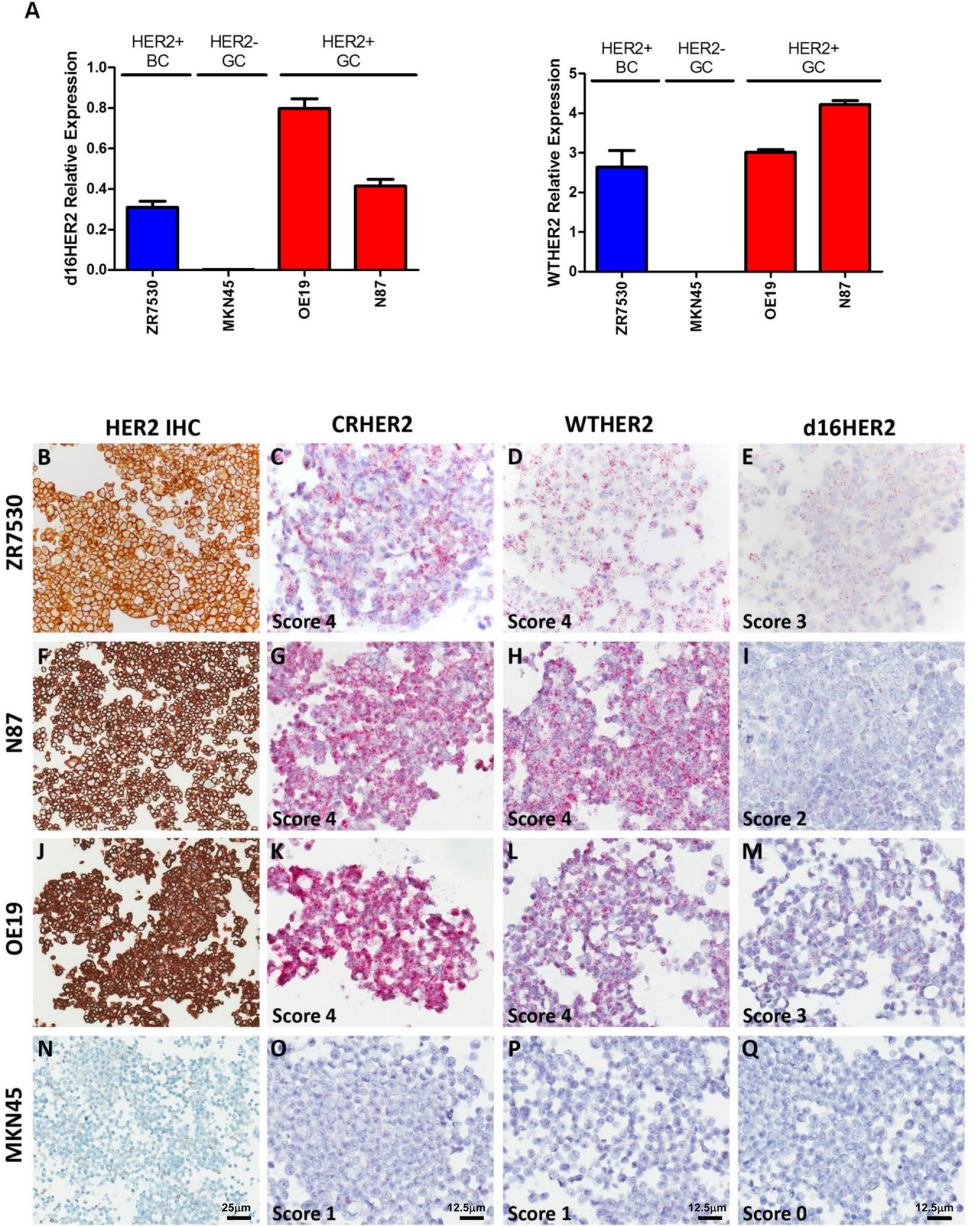
d16HER2 and WTHER2 isoform detection in HER2-positive BC (ZR75.30), GC (N87) and Oesophago-gastric (OGC) (OE19) and HER2-negative GC (MKN45) cell lines evaluated using qPCR, immunohistochemistry (IHC) and mRNA bright-field *in situ* hybridization (ISH) analyses. (**A**) qPCR analysis of relative d16HER2 and WTHER2 expression; (**B**–**Q**) HER2 expression by IHC and WTHER2 and d16HER2 isoforms detection by mRNA ISH. The ZR75.30 cell line (**B**–**E**) exhibits positive HER2 IHC reactivity (**B**); CRHER2 and WTHER2 mRNA ISH (score 4) (**C**,**D**); d16HER2 mRNA ISH (score 3) (**E**). The N87 cell line (**F**,**G**,**H**,**I**) exhibits positive HER2 IHC reactivity (**F**); CRHER2 and WTHER2 mRNA ISH (score 4) (**G**,**H**); d16HER2 mRNA ISH (score 2) (I). The OE19 cell line (**J**–**M**) exhibits positive HER2 IHC reactivity (**J**); CRHER2 and WTHER2 mRNA ISH (score 4) (**K**,**L**); d16HER2 mRNA ISH (score 3) (M). The MKN45 cell line (**N**,**O**,**P**,**Q**) exhibits negative HER2 IHC reactivity (N); CRHER2 and WTHER2 mRNA ISH (score 1) (**O**,**P**); d16HER2 mRNA ISH (score 0) (Q). Original magnification: 20 × (**B**,**F**,**J**,**N**; scale bar: 25 μm) and 40 × (**C**–**Q**; scale bar: 12,5 μm).

Then, we tested FFPE sections of different human commercial HER2-positive cell lines, such as ZR75.30, N87, and OE19, using qPCR (Fig. 1A), IHC (Fig. 1B,F,J; Supplementary Table S1) and mRNA ISH (Fig. 1C–M; Supplementary Table S1). As a negative control, the same analyses were performed on FFPE sections of the HER2-negative MKN45 GC cell line. Although both CRHER2 and WTHER2 probes yielded a score 4 for WTHER2 mRNA ISH in all HER2-positive cell lines (Fig. 1C–L; Supplementary Table S1), the d16HER2 probe exhibited moderate/high d16HER2 expression and yielded scores of 2 in N87 cells and 3 in ZR75.30 and OE19 cells (Fig. 1I,E,M; Supplementary Table S1). Such findings were confirmed by IHC (Fig. 1B,F,J; Supplementary Table S1) and qPCR (Fig. 1A). Scores of 0 and 1 were detected in MKN45 cells using the mRNA probe sets tested (Fig. 1O,P,Q; Supplementary Table S1), IHC (Fig. 1N*;* Supplementary Table S1) and qPCR (Fig. 1A).

### d16HER2 expression in the clinical setting

**Figure 2:**
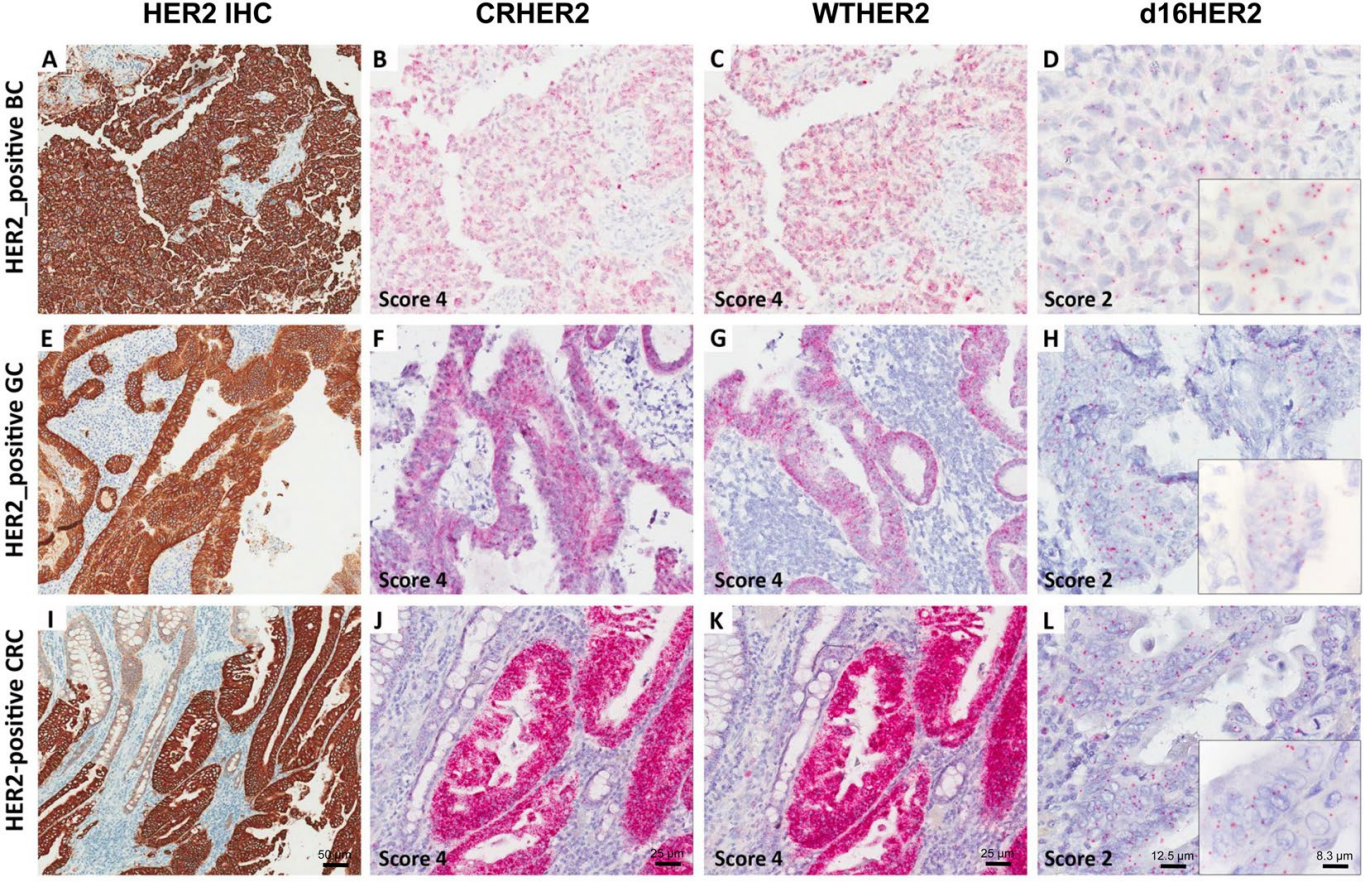
d16HER2 and WTHER2 isoform detection in human HER2-positive breast cancer (BC), gastric cancer (GC) and colorectal cancer (CRC) cases evaluated using immunohistochemistry (IHC) and mRNA bright-field *in situ* hybridization (ISH) analyses. The BC sample (No. 19 in Table 1) exhibits positive HER2 IHC reactivity (3+) (**A**); CRHER2 and WTHER2 mRNA ISH (score 4) (**B**,**C**); d16HER2 mRNA ISH (score 2) (**D**). The GC sample (No. 31 in Table 1) exhibits positive HER2 IHC reactivity (3+) (**E**); CRHER2 and WTHER2 mRNA ISH (score 4) (**F**,**G**); d16HER2 mRNA ISH (score 2) (**H**). The CRC sample (No. 42 in Table 1) exhibits positive HER2 IHC reactivity (3+) (**I**); CRHER2 and WTHER2 mRNA ISH (score 4) (**J**,**K**); d16HER2 mRNA ISH (score 2) (L). Original magnification: 10 × (**A**,**E**,**I**; scale bar: 50 μm), 20 × (**B**,**C**,**F**,**G**,**J**,**K**; scale bar: 25 μm), 40 × (**D**,**H**,**L**; scale bar: 12,5 μm) and insets 60 × (scale bar: 8,3 μm).

**Figure 3:**
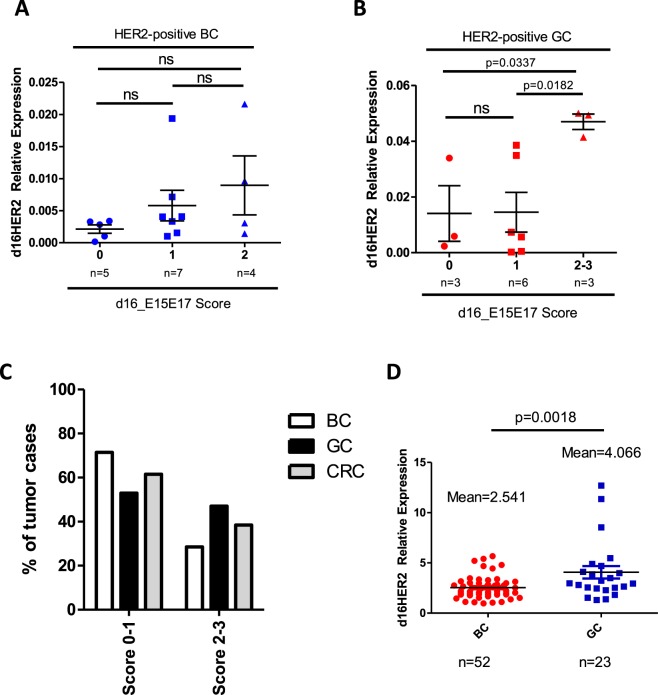
Association between relative d16HER2 expression in human HER2-positive breast cancer (BC) and gastric cancer (GC) frozen cases using qPCR analysis and d16HER2 mRNA bright-field *in situ* hybridization (ISH) scores evaluated in matched FFPE samples. (**A**) BC (n = 16) and (**B**) GC (n = 12) tissue samples. Significance was calculated by unpaired *t* test between score 2-3 *versus* score 1 and score 2-3 *versus* score 0 (p = 0.0182 and p = 0.0337, respectively). (**C**) Frequency of d16HER2 scores (from 0 to 3) in HER2-positive BC, GC and CRC FFPE samples. (**D**) Relative expression of d16HER2 (represented as ratio between d16HER2/WTHER2 transcripts) in human HER2-positive BC (n = 52) and GC (n = 23) frozen samples.

We then performed mRNA ISH analyses in FFPE tumor samples of HER2-positive BC (n = 21), GC (n = 17) and CRC (n = 13) patients that were already assessed using HER2 IHC and DNA ISH (Table 1). Matched d16HER2 transcript expression levels by qPCR were available only for breast and gastric cases due to lack of CRC frozen tissues (Table 1). Signals for d16HER2, WTHER2 and CRHER2 probes targeting their specific mRNAs were exclusively observed in epithelial cells. Normal epithelial cells occasionally expressed CRHER2 and WTHER2 albeit at very low levels. d16HER2 mRNA expression was exclusively observed in neoplastic cells. CRHER2 and WTHER2 probes provided inter-individually heterogeneous scores ranging from 1 to 4 in HER2-positive BC, GC and CRC (Table 1). Interestingly, cases with a HER2 IHC 3+ exhibited a score ≥ 3 for CRHER2, whereas cases with HER2 IHC 2 + and HER2 DNA ISH amplification exhibited greater score variability (1 to 4) (Table 1). Similar results were obtained for WTHER2 (Table 1). The d16HER2 probe allowed specific identification of the inter-individual variability of d16HER2 (score 0 to 3) (Table 1). A score of 3 was reported only in 5 cases (all gastrointestinal cancers: 4 GC and 1 CRC), whereas scores of 2 and 1 were noted in 13 and 21 cases, respectively. Figure 2B,C,F,G,J,K present examples of the 3 distinct tumor types (BC, GC and CRC) with scores of 4 for CRHER2 and WTHER2 probes and a score of 2 for the d16HER2 probe (Fig. 2D,H,L). As further proof of the suitability of the d16HER2 mRNA ISH score to quantify its expression levels in HER2-positive tumors, we observed a positive association between d16HER2 mRNA ISH score levels and qPCR values in human HER2-positive BC (Table 1; Fig. 3A) that became statistically significant in GC (Table 1, Fig. 3B; Score 2–3 *versus* Score 1: p = 0.0182; Score 2–3 *versus* Score 0: p = 0.0337). Even if the small samples sizes limit the comparison analyses among different histotypes, the analysis of d16HER2 expression through mRNA ISH revealed a numerically lower proportion of BC samples with scores of ≥2 (23%) compared with GC (47%) and CRC (38%) (Fig. 3C).

**Table 1:** Molecular features of human HER2-positive cancer samples with different histotypes.

No.	HER2 IHC* (score)	HER2 DNA ISH*	CRHER2 mRNA ISH* (score)	WTHER2 mRNA ISH* (score)	d16HER2 mRNA ISH* (score)	Relative d16HER2 Expression (2^-dct)^†^
**Breast Carcinoma**						
1	3+	nd	4	4	0	0,003274928
2	3+	nd	4	3	1	0,00714064
3	3+	nd	3	2	0	0,000151322
4	2+	Amplified	1	1	0	0,002828651
5	2+	Amplified	2	2	0	0,001001195
6	3+	Amplified	4	4	1	0,019366835
7	3+	nd	4	4	2	0,001424807
8	2+	Amplified	2	2	1	0,001004839
9	2+	Amplified	4	3	1	nd
10	3+	nd	4	4	1	nd
11	3+	nd	4	3	2	nd
12	3+	nd	4	4	1	0,021617642
13	3+	Amplified	4	3	1	0,0040731
14	3+	Amplified	4	3	1	0,003322517
15	3+	nd	3	2	2	0,003066318
16	3+	Amplified	4	4	2	0,009641344
17	3+	nd	4	4	1	0,004058702
18	3+	Amplified	4	4	0	0,003391902
19	3+	nd	4	4	2	nd
20	2+	Amplified	2	2	0	nd
21	3+	Amplified	3	2	1	0,001526385
**Gastric Carcinoma**						
22	3+	Amplified	4	4	1	0,0056482
23	3+	Amplified	4	3	2	nd
24	3+	Amplified	4	3	1	0,000224
25	3+	Amplified	4	4	0	0,0339334
26	3+	Amplified	4	4	0	0,0023472
27	3+	Amplified	4	4	1	0,038539
28	2+	Amplified	4	4	1	0,0073269
29	3+	Amplified	4	4	1	0,0348574
30	3+	nd	4	4	3	0,0500333
31	3+	Amplified	4	4	2	0,0493866
32	3+	Amplified	4	4	2	0,0414468
33	2+	Amplified	2	2	1	0,0004887
34	3+	Amplified	3	3	0	0,0058868
35	3+	Amplified	4	4	3	nd
36	3+	Amplified	4	4	3	nd
37	3+	Amplified	4	4	3	nd
38	3+	Amplified	4	4	2	nd
**Colon Carcinoma**						
39	3+	nd	4	4	1	nd
40	2+	Amplified	3	2	1	nd
41	2+	Amplified	4	3	1	nd
42	3+	nd	4	4	2	nd
43	3+	nd	4	4	3	nd
44	3+	nd	4	4	0	nd
45	3+	nd	4	4	2	nd
46	3+	nd	4	4	2	nd
47	2+	Amplified	3	2	1	nd
48	2+	Amplified	2	2	0	nd
49	3+	nd	4	4	1	nd
50	3+	Amplified	4	4	2	nd
51	2+	Amplified	3	2	0	nd

*Performed on formalin fixed paraffin-embedded tissue sections; ^†^performed on available frozen tissues.

IHC, immunohistochemistry; ISH, *in situ* hybridization; CR, common region; WT, WTHER2; d16, d16HER2; nd, not done.

Significantly increased d16HER2 expression (represented as ratio between d16HER2/WTHER2 transcripts) in GC *versus* BC (p = 0.0018) was also evident by qPCR results in terms of d16HER2 values (Fig. 3D).

### Assessment of d16HER2 expression in GC xenopatients treated with trastuzumab

**Figure 4:**
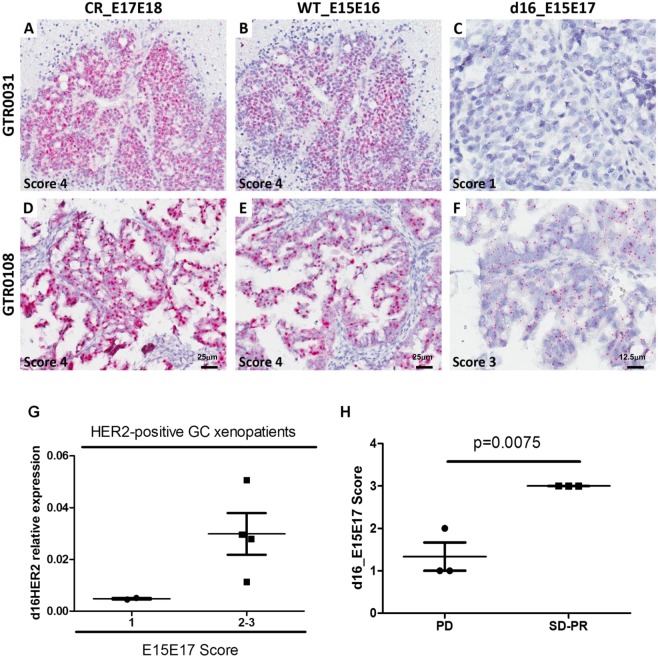
d16HER2 and WTHER2 isoform detection in two representative HER2-positive gastric cancer (GC) PDX models evaluated using mRNA bright-field *in situ* hybridization (ISH) and qPCR analyses. (**A**–**C**) GTR 0031 exhibits CRHER2 and WTHER2 mRNA ISH scores of 4 (**A**,**B**) and d16HER2 mRNA ISH score of 1 (**C**). (**D**–**F**) GTR 0108 exhibits CRHER2 and WTHER2 mRNA ISH scores of 4 (**D**,**E**) and d16HER2 mRNA ISH score of 3 (**F**). Original magnification: 20 × (**A**–**E**; scale bar: 25 μm) and 40 × (**C**,**F**; scale bar: 12,5 μm). (**G**) Association between relative d16HER2 expression in HER2-positive GC PDXs using qPCR analysis and d16HER2 mRNA bright-field ISH scores. (**H**) Association between d16HER2 ISH scores evaluated in matched FFPE HER2-positive GC samples and trastuzumab susceptibility. Significance was calculated by unpaired t test between PD versus SD/PR (p = 0.0075). CR: Complete response (tumor mass disappears); PR: partial response (>50% reduction of tumor volume); PD: progressive disease (>35% increase of tumor volume); SD: stable disease (intermediate response).

Finally, d16HER2 expression (scores of 1 to 3) was observed in 6 HER2-positive GC patient-derived xenografts (PDXs) (Fig. 4C,F) bearing reactivity for CRHER2 (Fig. 4A,D) and WTHER2 probes (Fig. 4B,E and Supplementary Table S2). As previously described for BC and GC samples, we observed an association between d16HER2 transcript levels assessed by ISH and qPCR analyses (Fig. 4G). Interestingly, xenotrials performed on the 6 HER2-positive PDXs revealed that only the 3 cases with a d16HER2 score of 3 achieved response following trastuzumab treatment (p = 0.0075). These results suggest that higher d16HER2 expression may reflect a condition of HER2 addiction and increased trastuzumab susceptibility (Fig. 4H).

### Clinical correlations

**Figure 5:**
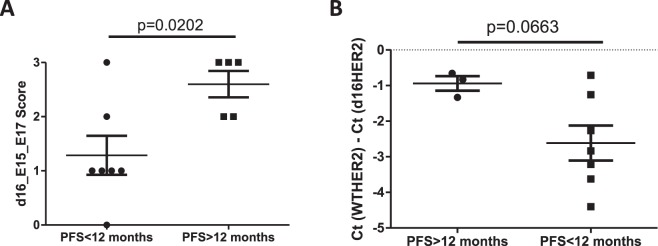
Association between d16HER2 mRNA bright-field ISH scores evaluated in HER2-positive GC FFPE samples (d16HER2 mRNA score) and PFS according to long (>12 months; n = 5) or short (<12 months; n = 7) times to trastuzumab response. Significance was calculated by unpaired *t* test (**A**). Association between abundance of d16HER2 *versus* WTHER2 transcript [Ct(WTHER2)-Ct(d16HER2)] evaluated in baseline plasma exosomes and PFS according to long (>12 months; n = 3) or short (<12 months; n = 7) times to trastuzumab response (**B**).

Given the results obtained in pre-clinical models and as proof-of-concept, we further investigated the clinical significance of d16HER2 as a potential surrogate biomarker of HER2 addiction and trastuzumab susceptibility (Fig. 5A,B). In light of the absence of d16HER2 reactivity with score >2 in our BC primary tumors cohort, here we report data on on gastrointestinal cancers. Among our GC cohort (n = 17), we selected 12 patients with metastatic disease and consequently treated with trastuzumab-based first-line treatment. Given that progression-free survival (PFS) exceeding 12 months for trastuzumab-based first-line treatment is only observed in < 10% of patients^2^, we considered such a time cut-off as clinically meaningful to define HER2 dependence. Of note, the 5 patients with PFS > 12 months had a significantly higher d16HER2 score than those with PFS < 12 months (p = 0.0202) (Fig. 5A). In particular, patients with PFS > 12 months were characterized by a d16HER2 score of 2 (n = 2) or 3 (n = 3), whereas all patients with shorter PFS had a score of 0 (n = 1), 1 (n = 4), 2 (n = 1) or 3 (n = 1) (Fig. 5A). As a further clinical tool to reveal and potentially track d16HER2 expression during trastuzumab-based treatment, we also tested a small-scale series of available baseline plasma samples obtained from HER2-positive metastatic GC patients prior to the start of trastuzumab-based first-line treatment (Fig. 5B). Again, d16HER2 expression was increased in circulating exosomes from long *versus* short trastuzumab responders (p = 0.0663) according to the above-mentioned clinical classification.

## Discussion

Given that d16HER2 overexpression/activation drives a key oncogenic signal in HER2-positive BC and is considered a “flag” of HER2 addiction and trastuzumab susceptibility^19,22^, we hypothesized that such a splice variant might play a crucial role also in other HER2-positive cancers, overall providing novel insights in the complex molecular landscape of HER2-driven tumors. Given the unavailability of in-house and customized specific monoclonal antibodies able to distinguish between the closely homologous d16HER2 variant and full-length WTHER2 molecules, and the inability to assess the abundance of the d16HER2 splice variant in the publicly TCGA breast cancer cohort due to lack of expression levels of the junction between exon 15 and exon 17 of ERBB2 (HER2) transcript, our aim was to develop experimental tools to detect and quantify d16HER2 expression in FFPE tumor tissues and patients’ blood, thereby facilitating clinical translation of d16HER2 as a biomarker in HER2-positive cancers, as demonstrated by BC pre-clinical data^19,22,24–26^.

The clinical relevance of mRNA ISH lies in the opportunity to detect RNA transcripts within individual neoplastic cells in archival FFPE tissues, thus providing a clear picture of the cellular distribution and potential tumor heterogeneity of HER2-positive tumors. On the other hand, mRNA measurement by qPCR is not easily transferrable in clinical settings given the requirements of frozen tumor specimens for proper transcript evaluation. In fact, the recovery of fresh tissue is not currently in the standard clinical management and the purification of mRNA could be affected by the presence of fibrotic, apoptotic and necrotic areas. On the opposite, FFPE represents the standard preservation procedure for diagnostic surgical pathology, mRNA ISH performed on this material could allow to score d16HER2 transcript expression in the same specimens used for the pathological diagnosis and assessment of HER2 expression/amplification. The capability of mRNA ISH to specifically discriminate between the constitutively expressed d16HER2 and WTHER2 isoforms was also confirmed by parallel qPCR testing of HER2-positive cell lines derived from both BC and GC/OGC. In addition to confirming the expected correlation between IHC and WTHER2 mRNA ISH scores, we also correlated d16HER2 mRNA ISH scores with qPCR measurements available for BC and GC, demonstrating the accuracy of our technique for scoring d16HER2 mRNA in FFPE tumor samples.

Given that d16HER2 overexpression/activation drives a key oncogenic signal in HER2-positive BC and is considered a “flag” of HER2 addiction and trastuzumab susceptibility^19,22,23^, we hypothesized that such splice variant might play a crucial role also in other HER2-positive cancers, overall providing novel insights in the complex molecular landscape of HER2-driven tumors. Specifically, our experiments were performed not only in HER2-positive BC and GC patients, for whom anti-HER2 treatments are currently recommended^1,2^, but also in CRC, where novel dual anti-HER2 combinations, such as trastuzumab + lapatinib or trastuzumab + pertuzumab, are considered highly promising in heavily pretreated patients^3,12^.

Our results clearly demonstrate the presence of outliers with high d16HER2 mRNA scores restricted to HER2-positive gastrointestinal malignancies coupled with increased d16HER2 expression compared with BC. Of note, experiments performed on preclinical models represented by primary GC PDXs suggest that increased d16HER2 expression is associated with clinical benefit/response to single-agent trastuzumab. Therefore, as previously shown in BC models^19^, our data provide evidence that d16HER2 may also be considered as a “flag” of HER2 addiction in GC as supported by the significant association we found between high d16HER2 score and prolonged PFS, and could be clinically investigated as a marker of trastuzumab susceptibility in several HER2-driven cancers, including CRC. In light of trastuzumab capability to impair cancer stem cell subpopulation in HER2-positive GC^30^ and our reported data in HER2-positive BC models^19,22^, we speculate that d16HER2 variant can have a key role also in HER2-positive GC stemness, though we are aware that functional experiments are mandatory to validate our hypothesis. As final proof-of-concept, we demonstrate that high d16HER2 mRNA scores are exclusively found in patients with long-term benefit from trastuzumab exceeding 12 months (clinical “outliers”), and d16HER2 expression is also increased in circulating tumor-released exosomes obtained from baseline plasma samples of long-term responders. This finding indicates that the definition of optimal cut-offs of liquid biopsy assays are needed for larger prospective cohorts. In this context, d16HER2-positive tumor released exosomes might represent an alternative tool to monitor the changes in tumor clonal composition allowing a dynamic treatment stratification^28,29^.

Our study has some limitations. First, due to the small number of tested samples, our results require broader validation within larger cohorts. In particular, we could not investigate the association of d16HER2 with initial clinico-pathological characteristics and patients’ outcomes. However, data obtained in PDXs suggest a positive correlation between the expression of d16HER2 and response to trastuzumab. Indeed, treatment of HER2-positive PDXs with trastuzumab resulted in a RECIST-like response only in the 3 models with tumors with high d16HER2 scores. Another limitation of our study is that we could not investigate the concordance between primary tumors and their metastases as well as HER2 modulation during treatment.

Regarding future applications of d16HER2 as a biomarker, we propose a validation of its accuracy for predicting HER2 dependence and long-term response from trastuzumab-based treatment in larger cohorts or for post-hoc translational analyses of pivotal trials. Regarding GC, better patient selection, including the selection of both positive (by HER2 copy numbers and/or d16HER2) and negative (by excluding tumors with co-existing oncogenic drivers)^11^ patients, could lead to a reassessment of the role of BC blockbuster drugs that failed in this disease, such as dual blockade with trastuzumab plus pertuzumab, TD-M1 and lapatinib, in highly HER2-addicted GC patients^31–33^. Regarding CRC, the landscape of primary or acquired resistance to dual HER2 blockade was described very recently^5^, and d16HER2 could implement such data by identifying the long-term responders described in the HERACLES trial^3^.

Given that our group and others have reported translational findings on the roles of d16HER2 in HER2-positive BC^18,19,22,24–26^, we firmly believe that an assessment of d16HER2 in clinical samples could help to identify tumors driven to HER2 signals and that are likely to respond to a trastuzumab/HER2 dual blockade, thus improving the management of HER2-positive disease.

## Materials and Methods

### Tumor cell lines

Human BC MCF7 (HER2-negative) and ZR75.30 (HER2-positive),and GC NCI-N87 (N87; HER2-positive) cell lines were purchased from ATCC (Rockville, MD, USA); human OGC OE19 cell line was purchased from ECACC General Cell Collection (Sigma–Aldrich, St. Louis, MO); and human MKN45 (HER2-negative) cell line was purchased from HSRRB (Health Science Research Resources Bank, Osaka, Japan). All tumor cell lines were grown as monolayer cultures in RPMI 1640 (EuroClone, Pero, MI, Italy) with 10% fetal bovine serum (FBS) and gentamycin (40 µg/ml). The genetic identity of the GC cell lines have been verified at Candiolo Cancer Institute (last verification December 2017) by STR DNA fingerprinting, using the Promega, Powerplex 16HS system according to manufacturer’s instructions (Cell ID, Promega, Madison, WI, USA). The STR profiles were compared to known ATCC fingerprints (ATCC.org), and to the Cell Line Integrated Molecular Authentication database (CLIMA) version 0.1.200808 (http://bioinformatics.istge.it/clima/). Human BC cell lines were authenticated by short tandem repeat DNA fingerprinting using the AmpFISTR identifier PCR Amplification Kit (Thermo Fisher, Waltham, MA, USA) yearly (last verification, November 2017).

Lentivirus-infected MCF7_d16 and MCF7_WT cell lines were engineered and grown as described^22^. All tumor cell lines were cultured at 37 °C in a humidified 5% CO_2_ atmosphere and routinely tested for mycoplasma contamination.

### Clinical samples

Fifty-one HER2-positive tumor FFPE samples, including BC (n = 21), GC (n = 17) and CRC (n = 13), were included in this study (Table 1). Patients’ clinical and pathological information were detailed in Supplementary Table S3, S4 and S5. HER2 positivity was centrally assessed by means of both immunohistochemistry (IHC) and ISH by two independent observers according to standard criteria and international guidelines^34–36^.

Fresh-frozen tumor tissues were available only for BC (n = 52) and GC (n = 23) samples for qPCR testing (Fig. 3D), and the fresh frozen counterparts of some of FFPE tissues were also included (see Table 1). These samples were therefore tested for the expression of d16HER2 and WTHER2 mRNA by qPCR, whereas plasma samples were available for exosomes isolation only for HER2-positive GC patients (n = 10). All procedures were in accordance with the Helsinki Declaration. Biospecimens used for research consisted of leftover material of samples collected during standard surgical and medical approaches at Fondazione IRCCS Istituto Nazionale dei Tumori of Milan and at the Azienda Ospedaliero-Universitaria Pisana, Pisa, Italy. Samples were donated by patients to the Institutional BioBanks for research purposes, and aliquots were allocated to this study after approval by the Institutional Review Board and a specific request to the Independent Ethical Committee of the institutes.

### HER2 IHC and ISH

To detect both WTHER2 and d16HER2 isoform expression, we used the anti-human c-erbB-2 A0485 polyclonal antibody (dilution 1:500; Dako) on 3-μm FFPE sections. IHC was performed using a BenchMark Ultra Platform (Ventana Medical Systems, Tucson, AZ) with the Optiview DAB Detection Kit (Ventana Medical Systems). HER2 immunoreactivity in breast and gastric carcinomas was scored as 0, 1+, 2+, 3+ according to international guidelines systems^34,37,38^. HER2 immunoreactivity in colon carcinomas was evaluated according to guidelines for gastric carcinomas.

Dual color silver *in situ* hybridization (DC-SISH) was performed on a BenchMark Ultra Platform (Ventana Medical Systems) according to the manufacturer’s protocol. The gene status was assessed on 3-μm FFPE sections using the INFORM HER2 Dual ISH DNA Probe Cocktail (Ventana Medical Systems). HER2 gene amplification was evaluated according to previously described scoring systems^35,37,38^. Briefly, HER2 gene amplification was defined as positive when HER2/CEP17 ratio was >2 or when HER2 gene copy number was of >6 for breast carcinomas or of >6 for gastric and colon carcinomas.

Scoring systems for HER2 protein expression and gene amplification evaluations were applied for clinical samples, cell lines and PDX.

### Xenograft transplantation experiments

HER2-positive GC samples were obtained from patients undergoing surgery in the following Italian Hospitals: Candiolo Cancer institute- FPO, IRCCS (Torino); San Giovanni Battista Hospital (Torino); Humanitas-IRCCS (Milano); and Treviglio-Caravaggio Hospital (Bergamo). All patients provided informed consent. Samples were obtained, and the study was conducted under the approval of the Review Boards of all the institutions. All the samples were de-identified before shipping to Candiolo Cancer Institute-FPO, IRCCS (Torino). The generation of PDX was conducted as previously reported^39^. GC material from San Giovanni Battista Hospital and Candiolo Cancer institute- FPO, IRCCS (Torino) was collected in medium 199 (Sigma–Aldrich) supplemented with 100 μg/mL levofloxacin stored at 4 °C and received within a few hours from surgical resection. GC material from other Italian Hospitals was collected in IGL-1 preservation medium (Pharmanovia, Jægersborg, Denmark) supplemented with 100 μg/mL levofloxacin, stored at 4 °C and received within 24 hours from surgical resection. At the Candiolo Cancer Institute, each sample was cut into 25- to 50-mm^3^ pieces in antibiotic-containing 199 medium. Some of the pieces were incubated overnight in RNAlater (Thermo Fisher) and then frozen at −80 °C for molecular analyses. One piece was fixed in formalin and centrally scored for HER2 expression level (HercepTest, Dako Denmark A/S, Glostrup, Denmark). All of the 6 GCs included in the study received a score of 3+. Two pieces were coated in Matrigel (BD Biosciences, Franklin Lakes, New Jersey, NJ, USA) and subcutaneously implanted in two or three different 4- to 6-week-old female NOD/SCID mice (Charles River, Calco, CO, Italy). After mass formation, tumors were passaged and expanded for at least 2 generations. The genetic identity between the original tumor and the corresponding PDX was evaluated by short tandem repeat profiling (Cell ID, Promega, Madison, WI, USA). An HER2 score of 3+ was confirmed on PDX using HercepTestTM. Tumor size was evaluated by caliper measurements. The approximate mass volume was calculated using the formula 4/3π.(*d*/2)2.*D*/2, where *d* is the minor tumor axis, and *D* is the major tumor axis. Established tumors (average volume 300 mm^3^) were treated for 6 weeks with 30 mg/Kg humanized monoclonal antibody trastuzumab via weekly intraperitoneal injection. Tumor response evaluation was performed using the following RECIST-like criteria: complete response (CR), tumor mass disappears; partial response (PR), >50% reduction of tumor volume; progressive disease (PD), >35% increase of tumor volume; and stable disease (SD), intermediate responses. All experiments were performed in accordance with relevant guidelines and regulations. All animal procedures were approved by the Ethical Commission of the Candiolo Cancer Institute (Candiolo, Torino, Italy) and the Italian Ministry of Health.

### mRNA bright-field ISH

In light of the almost identical DNA sequence between WTHER2 and d16HER2 (Supplementary Fig. 1A), we properly used mRNA bright-field ISH coupled with qPCR analysis for both transcripts^19,22^. Three different probes for HER2 were used in the study: BA-Hs-ERBB2-E15E17 recognizes the exon junction between exons 15 and 17 targeting the d16HER2 splice variant mRNA (d16HER2); BA-Hs-ERBB2-E15E16 recognizes the exon junction between exons 15 and 16 targeting WTHER2 mRNA (WTHER2) and BA-Hs-ERBB2-E17E18 recognizes the exon junction between exons 17 and 18 targeting a common region both in d16HER2 and WTHER2 mRNA (CRHER2) (Advanced Cell Diagnostics, Inc., Hayward, CA) (Supplementary Fig. 1B–D). Each tested case included both a positive control probe (BA-Hs-PPIB-1ZZ probe that recognizes peptidylprolyl isomerase B mRNA) for evidence of RNA quality and a negative control probe (BA-DapB-1ZZ probe that recognizes the bacterial gene dihydrodipicolinate reductase).

ISH was manually performed using the Basescope – Red assay (Advanced Cell Diagnostics) according to the manufacturer’s instructions. Briefly, 5-µm deparaffinized FFPE tissue sections were incubated with hydrogen peroxide for 10 minutes at room temperature, boiled with RNAscope 1X Target Retrieval Reagent (Advanced Cell Diagnostics) at 99 °C for 15 minutes using a steamer (Braun Multiquick FS-20 Steamer, Kronberg im Taunus, Germany), and incubated with RNAscope Protease III for 30 minutes (clinical samples) or 20 minutes (cell lines and xenografts) at 40 °C. Tissue sections were then hybridized for 2 hours at 40 °C with the target probes or control probes and sequentially incubated with Amp0 to Amp6 reagents. After signal detection with Fast-Red A and Fast-Red B, slides were counterstained with hematoxylin and coverslipped with VectaMount mounting medium (Vector Laboratories, Inc., Burlingame, CA). All incubations were performed at 40 °C in an oven (HybEZ Oven, Advanced Cell Diagnostics). Each tested case was considered to be evaluable only when the quality of the mRNA as evaluated by the positive control probe was good in a representative area of the sample, and no signal (or <1 dot every 20 cells) was observed with the negative control probe. Positive staining signals were identified as red, punctuate dots or clusters (i.e., numerous overlapping mRNA signals that cannot be individually counted) present in the cytoplasm and/or nucleus. Samples were evaluated with a scoring system that categorizes cases into five grades according to the number of dots visualized under a bright-field microscope following the manufacturer’s recommendations. In brief, the following scoring system was used: score 0, no staining or less than 1 dot for every 20 cells; score 1, 1 dot/cell; score 2, 2-3 dots/cell and no or very few clusters; score 3, 4-10 dots/cell and less than 10% cells with clusters; score 4, >10 dots/cell and greater than 10% positive cells with clusters. All slides (d16HER2, WTHER2 and CRHER2 mRNA ISH analyses) were scored independently by 2 readers during two different temporal sessions (15 days apart). All people involved in microscopic slides review were blinded from the results of all tested assays and the scoring of the other reviewer. Discrepant evaluations were reviewed and resolved by consensus interpretation.

### qPCR

Human tumor cell lines (n = 7); HER2-positive BC (n = 52), GC (n = 23) and GC PDX models (n = 6); and frozen primary and metastatic tumor samples were analyzed by qPCR to determine d16HER2 transcript levels. Briefly, total RNA from human tumor tissues was extracted using the ReliaPrep miRNA Cell and Tissue Miniprep System kit (Promega, Fitchburg, WI, USA) following the manufacturer’s instructions. cDNAs were reverse-transcribed from 1 µg of total RNA in a 20-µl volume using the High-Capacity RNA-to-cDNA Kit (Thermo Fisher Scientific, Waltham, Massachusetts, USA), and 25 ng cDNA was examined by qPCR using the Applied Biosystems *SYBR Green* dye-based PCR assay on the ABI Prism 7900HT sequence detection system (Applied Biosystems, Foster City, CA). The d16HER2 variant was amplified using 200 nM primers^26^. mRNA from the murine mammary carcinoma cell line (MI6) generated in house from a spontaneous mammary lesion grown in mice transgenic for the d16HER2 splice variant^19,22^ was used as positive control. Data were normalized to GAPDH. The relative abundance of d16HER2 mRNA was calculated using the comparative delta Ct method^40^.

### Isolation of exosomes from plasma samples and qPCR analyses

Exosomes were purified from 1 ml of plasma derived from 10 HER2-positive GC patients and were isolated using ExoQuick-TC exosome precipitation solution (System Biosciences, Mountain View, CA, USA) according to the manufacturer’s guidelines. Total RNA from exosomes was isolated using the ReliaPrep miRNA Cell and Tissue Miniprep System kit (Promega, Fitchburg, WI, USA). cDNA was reverse-transcribed from 100 ng of total RNA in a 20-µl volume using the High-Capacity RNA-to-cDNA Kit (Thermo Fisher Scientific, Waltham, MA, USA), and 10 ng of cDNA was examined by qPCR using Applied Biosystems *SYBR Green* dye-based PCR assays on the ABI Prism 7900HT sequence detection system (Applied Biosystems, Foster City, CA). d16HER2 and WTHER2 transcripts were amplified using 200 nM primers^26^. The relative abundance of d16HER2 mRNA compared with WTHER2 was calculated using the comparative *C*t method^40^, and d16HER2 transcript levels were indicated as *C*t (d16HER2) - *C*t (WTHER2).

### Significance

Differences between groups were tested using a two-tailed unpaired t-test. Differences were considered significant at p < 0.05.

## Supplementary information

Supplementary Informations
