# Author Correction: The landscape of d16HER2 splice variant expression across HER2-positive cancers

**DOI:** 10.1038/s41598-020-63942-4

**Published:** 2020-04-24

**Authors:** Chiara Costanza Volpi, Filippo Pietrantonio, Annunziata Gloghini, Giovanni Fucà, Silvia Giordano, Simona Corso, Giancarlo Pruneri, Maria Antista, Chiara Cremolini, Elena Fasano, Serena Saggio, Simona Faraci, Maria Di Bartolomeo, Filippo de Braud, Massimo Di Nicola, Elda Tagliabue, Serenella Maria Pupa, Lorenzo Castagnoli

**Affiliations:** 1 Department of the Pathology and Laboratory Medicine, Fondazione IRCCS Istituto Nazionale dei Tumori, Milan, Italy; 2 Department of Oncology and Hemato-oncology, Fondazione IRCCS Istituto Nazionale dei Tumori, Milan, Italy; 3 Faculty of Medicine, Oncology and Hemato-Oncology Department, Università degli Studi di Milano, Milan, Italy; 4 Department of Oncology, University of Turin, Turin, Italy; 5 Unit of Medical Oncology, Azienda Ospedaliera-Universitaria Pisana, Pisa, Italy; 6 Molecular Targeting Unit, Department of Research, Fondazione IRCCS Istituto Nazionale dei Tumori, Milan, Italy; 7 Candiolo Cancer Institute, FPO - IRCCS - Candiolo (TO), Turin, Italy

Correction to: *Scientific Reports* 10.1038/s41598-019-40310-5, published online 05 March 2019

The original version of this Article omitted an affiliation for Silvia Giordano and Simona Corso. The correct affiliations for Silvia Giordano and Simona Corso are listed below:

Department of Oncology, University of Turin, Turin, Italy

Candiolo Cancer Institute, FPO - IRCCS - Candiolo (TO), Turin, Italy

This has now been corrected in the HTML and PDF versions of this Article, and in the accompanying Supplemental Material.

