# Supplementary material for: The landscape of d16HER2 splice variant expression across HER2-positive cancers
Source: Sci Rep. 2019 Mar 5;9:3545. doi: 10.1038/s41598-019-40310-5 (PMC6401102; doi:10.1038/s41598-019-40310-5)
Supplement: Supplementary file 1 — Supplementary Informations [file 41598_2019_40310_MOESM1_ESM.pdf]

Volpi et al, Supplementary Figure 1

The landscape of d16HER2 splice variant expression across HER2-positive cancers

Chiara Costanza Volpi[1], Filippo Pietrantonio[2,3], Annunziata Gloghini[1], Giovanni Fucà[2], Silvia Giordano[4], Simona Corso[4], Giancarlo Pruneri[1], Maria Antista[2], Chiara Cremolini**[5]**, Elena Fasano[6], Serena Saggio[2], Simona Faraci[6], Maria Di Bartolomeo[2], Filippo de Braud[2,3], Massimo Di Nicola[2], Elda Tagliabue[6], Serenella Maria Pupa[6] and Lorenzo Castagnoli[6]

CCV[1] and FP[2,3] contributed equally.
SMP[6] and LC[6] contributed equally.

Volpi et al, Supplementary Figure 1

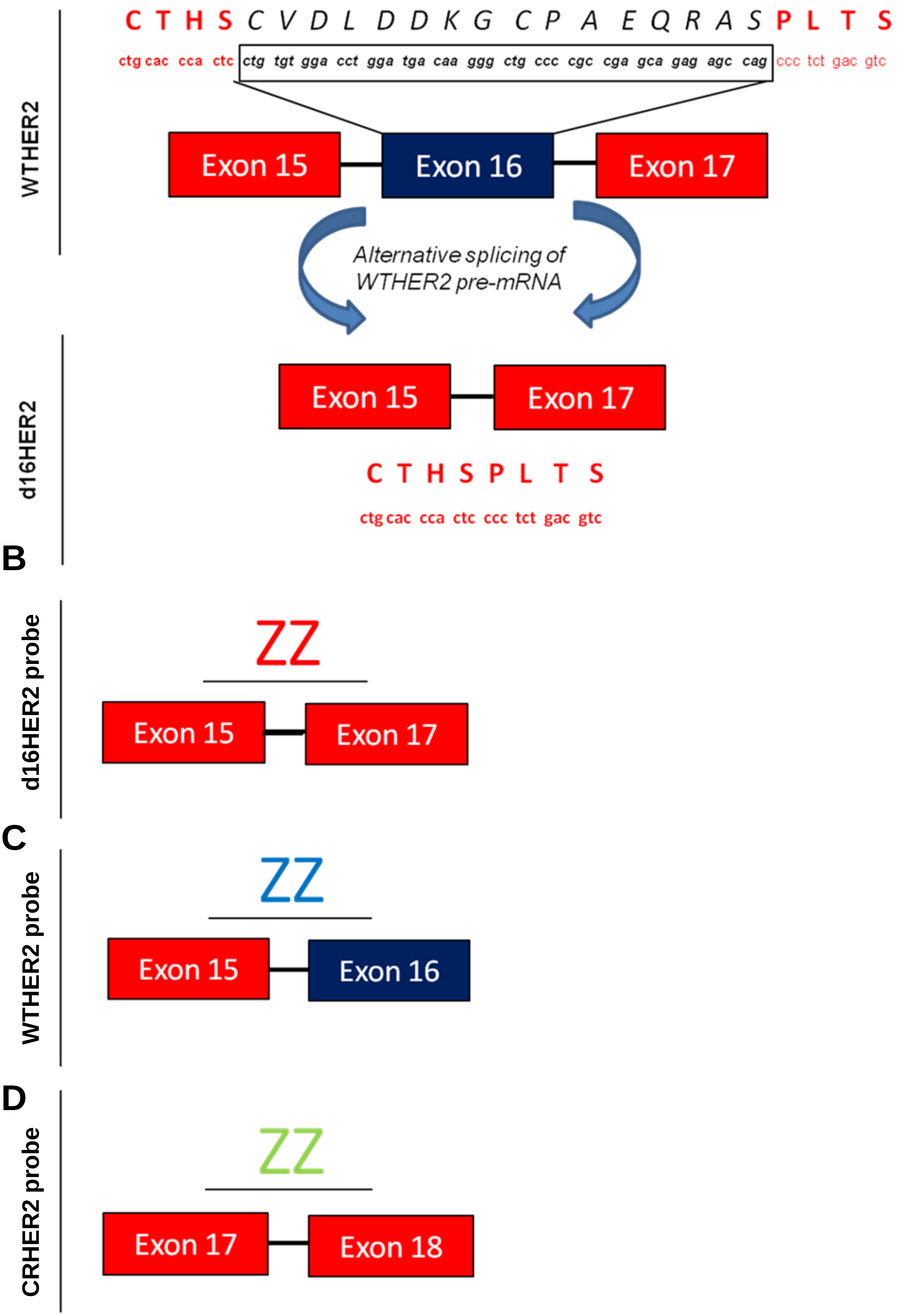

Volpi et al, Supplementary Figure 2

The landscape of d16HER2 splice variant expression across HER2-positive cancers

Chiara Costanza Volpi[1], Filippo Pietrantonio[2,3], Annunziata Gloghini[1], Giovanni Fucà[2], Silvia Giordano[4], Simona Corso[4], Giancarlo Pruneri[1], Maria Antista[2], Chiara Cremolini[5], Elena Fasano[6], Serena Saggio[2], Simona Faraci[6], Maria Di Bartolomeo[2], Filippo de Braud[2,3], Massimo Di Nicola[2], Elda Tagliabue[6], Serenella Maria Pupa[6] and Lorenzo Castagnoli[6]

CCV[1] and FP[2,3] contributed equally.
SMP[6] and LC[6] contributed equally.

Volpi et al, Supplementary Figure 2

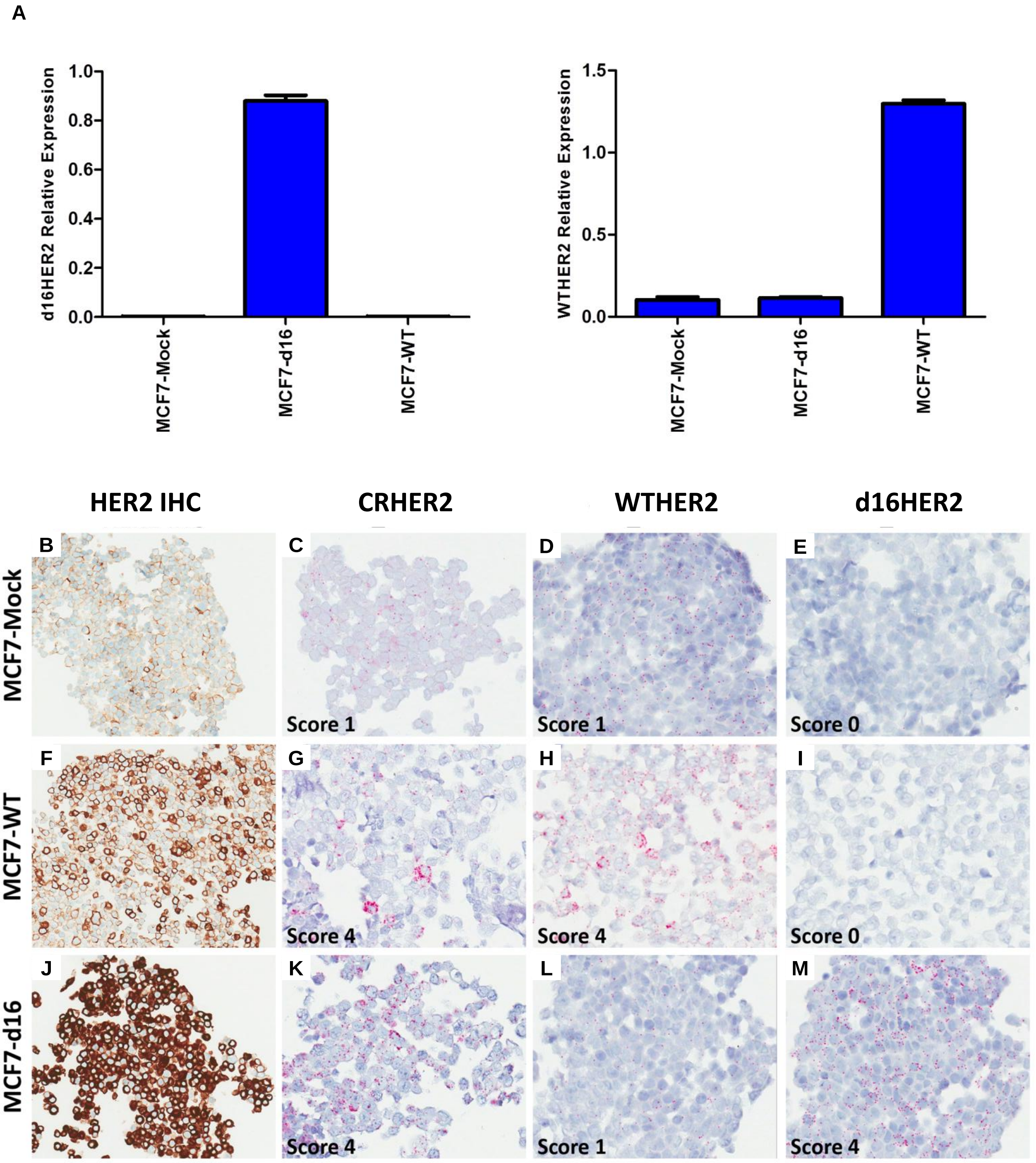

The landscape of d16HER2 splice variant expression across HER2-positive cancers

Chiara Costanza Volpi[1], Filippo Pietrantonio[2,3], Annunziata Gloghini[1], Giovanni Fucà[2], Silvia Giordano[4], Simona Corso[4], Giancarlo Pruneri[1], Maria Antista[2], Chiara Cremolini[5], Elena Fasano[6], Serena Saggio[2], Simona Faraci[6], Maria Di Bartolomeo[2], Filippo de Braud[2,3], Massimo Di Nicola[2], Elda Tagliabue[6], Serenella Maria Pupa[6] and Lorenzo Castagnoli[6]

CCV[1] and FP[2,3] contributed equally.

SMP[6] and LC[6] contributed equally.

Supplementary Fig. 1. Exons and final transcripts of full-length/WTHER2 and d16HER2 isoforms. (**A**) Schematic representation of the alternative d16HER2 splice variant derived by the processing of WTHER2 pre-mRNA; (**B**), (**C**) and (**D**) mRNA ISH probes targeting d16HER2 transcript (d16HER2), WTHER2 transcript (WTHER2) and a common region for both HER2 isoform transcripts (CRHER2), respectively.

Supplementary Fig. 2. Ectopic expression of d16HER2 and WTHER2 isoforms in properly engineered MCF7-d16, MCF7-WT and MCF7-Mock infected cells evaluated using qPCR, immunohistochemistry (IHC) and mRNA bright-field in situ hybridization (ISH) analyses. (**A**) qPCR analysis of relative d16HER2 and WTHER2 expression; (**B, F, J**) HER2 expression by IHC: MCF7-Mock exhibits negative HER2 IHC reactivity (B); the MCF7-WT exhibits HER2 IHC reactivity in 70% of cells (F) and MCF7-d16 exhibits positive HER2 IHC reactivity (J). (**C, D, E, G, H, I, K, L, M**) WTHER2 and d16HER2 isoforms detection by mRNA ISH: CRHER2 and WTHER2 mRNA ISH (score 1) (C, D); d16HER2 mRNA ISH (score 0) (E) in MCF-7-Mock. CRHER2 and WTHER2 mRNA ISH (score 4) (G, H); d16HER2 mRNA ISH (score 0) (I) in MCF7-WT. CRHER2 mRNA ISH (score 4) (K); WTHER2 mRNA ISH (score 1) (L); d16HER2 mRNA ISH (score 4) (M) in MCF7-d16. Original magnification: 20X (B, F, J; scale bar: 25 μm) and 40X (C, D, E, G,, H, I, K, L, M, N; scale bar: 12,5 μm).

The landscape of d16HER2 splice variant expression across HER2-positive cancers

Chiara Costanza Volpi[1], Filippo Pietrantonio[2,3], Annunziata Gloghini[1], Giovanni Fucà[2], Silvia Giordano[4], Simona Corso[4], Giancarlo Pruneri[1], Maria Antista[2], Chiara Cremolini[5], Elena Fasano[6], Serena Saggio[2], Simona Faraci[6], Maria Di Bartolomeo[2], Filippo de Braud[2,3], Massimo Di Nicola[2], Elda Tagliabue[6], Serenella Maria Pupa[6] and Lorenzo Castagnoli[6]

CCV[1] and FP[2,3] contributed equally.

SMP[6] and LC[6] contributed equally.

Supplementary Table S1. Histological and molecular features of human cancer cell lines

Cell Line	HER2 IHC score	CRHER2 score	WTHER2 score	d16HER2 score
MCF7-Mock	1+	1	1	0
MCF7-WT	3+	4	4	0
MCF7-d16	3+	4	1	4
ZR75.30	3+	4	4	3
N87	3+	4	4	2
OE19	3+	4	4	3
MKN45	0	1	1	0

The landscape of d16HER2 splice variant expression across HER2-positive cancers

Chiara Costanza Volpi[1], Filippo Pietrantonio[2,3], Annunziata Gloghini[1], Giovanni Fucà[2], Silvia Giordano[4], Simona Corso[4], Giancarlo Pruneri[1], Maria Antista[2], Chiara Cremolini[5], Elena Fasano[6], Serena Saggio[2], Simona Faraci[6], Maria Di Bartolomeo[2], Filippo de Braud[2,3], Massimo Di Nicola[2], Elda Tagliabue[6], Serenella Maria Pupa[6] and Lorenzo Castagnoli[6]

CCV[1] and FP[2,3] contributed equally.

SMP[6] and LC[6] contributed equally.

Supplementary Table S2. Molecular features of human HER2-positive GC xenografts.

Xenopatient	CRHER2 mRNA ISH score	WTHER2 mRNA ISH score	d16HER2 mRNA ISH score
SG 55	4	4	3
SG 66	4	4	2
GTR 0031	4	4	1
GTR 0108	4	4	3
GTR 0109	3	3	1
GTR 0233	4	4	3

The landscape of d16HER2 splice variant expression across HER2-positive cancers

Chiara Costanza Volpi[1], Filippo Pietrantonio[2,3], Annunziata Gloghini[1], Giovanni Fucà[2], Silvia Giordano[4], Simona Corso[4], Giancarlo Pruneri[1], Maria Antista[2], Chiara Cremolini[5], Elena Fasano[6], Serena Saggio[2], Simona Faraci[6], Maria Di Bartolomeo[2], Filippo de Braud[2,3], Massimo Di Nicola[2], Elda Tagliabue[6], Serenella Maria Pupa[6] and Lorenzo Castagnoli[6]

CCV[1] and FP[2,3] contributed equally.

SMP[6] and LC[6] contributed equally.

Supplementary Table S3. Clinical and pathological information of BC cases.

No.	Age	Sex	Histotype	Grade	pTNM	ER% IHC	PgR% IHC	Relapse
**1**	42	F	Ductal	3	pT2 pN0	0-1	0-1	No
**2**	61	F	Ductal	3	pT2 pN3a	0-1	10-33	No
**3**	59	F	Ductal	3	pT3 pN3a	66-100	10-33	No
**4**	43	F	Ductal	3	pT1c pN0	0-1	0-1	No
**5**	50	F	Lobular	2	pT1c pN1a(sn)	66-100	66-100	No
**6**	46	F	Ductal	3	pT2 pN1a	33-66	0-1	Yes
**7**	70	F	Ductal	3	pT1c pN0	0-1	0-1	No
**8**	67	F	Ductal	3	pT2 pN2a	0-1	0-1	Yes
**9**	32	F	Ductal	2	pT1c pN0	1-10	1-10	Yes
**10**	44	F	Ductal	2	pT1c pN1a(sn)	66-100	66-100	No
**11**	59	F	Ductal	3	pT2 pN2a	0-1	0-1	Yes
**12**	46	F	Ductal	3	pT1c pN1mi	33-66	10-33	No
**13**	50	F	Ductal	3	pT2 pN3a	66-100	33-66	No
**14**	48	F	Ductal	2	pT1c pN1mi(sn)	66-100	66-100	No
**15**	56	F	Ductal	3	pT1c pN3a	0-1	0-1	No
**16**	47	F	Ductal	3	pT3 pN3a	0-1	0-1	No
**17**	58	F	Ductal	3	pT2 pN1a	0-1	0-1	No
**18**	34	F	Ductal	2	pT1a pN0	0-1	0-1	No
**19**	57	F	Ductal	3	pT2 pN2a	0-1	0-1	No
**20**	50	F	Lobular	2	pT4a pN0(sn)	66-100	1-10	No
**21**	54	F	Ductal	3	pT2 pN1a	66-100	33-66	Yes

The landscape of d16HER2 splice variant expression across HER2-positive cancers

Chiara Costanza Volpi[1], Filippo Pietrantonio[2,3], Annunziata Gloghini[1], Giovanni Fucà[2], Silvia Giordano[4], Simona Corso[4], Giancarlo Pruneri[1], Maria Antista[2], Chiara Cremolini[5], Elena Fasano[6], Serena Saggio[2], Simona Faraci[6], Maria Di Bartolomeo[2], Filippo de Braud[2,3], Massimo Di Nicola[2], Elda Tagliabue[6], Serenella Maria Pupa[6] and Lorenzo Castagnoli[6]

CCV[1] and FP[2,3] contributed equally.

SMP[6] and LC[6] contributed equally.

Supplementary Table S4. Clinical and pathological information of GC cases

No.	Age	Sex	Site of origin	Histotype	Grade	Primary tumor resected	pTNM	Date of metastases	synchronous/ metachronous
**22**	66	M	GC	Intestinal	3	Y	pT1b pN3	30/01/2018	M
**23**	72	M	GC	Intestinal	3	N	na	13/10/2016	S
**24**	67	M	GC	Intestinal	3	Y	pT2 pN1	na	na
**25**	59	M	GC	Intestinal	3	N	na	31/03/2014	S
**26**	75	M	GC	Intestinal	3	Y	pT3 pN2	na	na
**27**	66	M	GC	Intestinal	2	Y	pT3pN0	na	na
**28**	67	F	GEJ	Mixed	3	Y	pT3 pN2	na	S
**29**	80	F	GC	Mixed	3	Y	pT4a pN3b	22/03/2017	S
**30**	79	M	GC	Intestinal	3	Y	pT4a pN2	01/09/2014	S
**31**	62	M	GEJ	Mixed	3	Y	pT3 pN3	23/01/2017	S
**32**	51	M	GC	Intestinal	3	N	na	06/10/2016	S
**33**	60	M	GC	Intestinal	1	Y	pT2 pN0	30/11/2012	M
**34**	68	M	GC	Intestinal	2	Y	pT1a pN0	na	na
**35**	41	M	GEJ	Intestinal	2	N	na	14/04/2014	S
**36**	45	M	GC	Intestinal	2	N	na	12/10/2012	S
**37**	67	M	GC	Intestinal	2	N	na	01/05/2013	S
**38**	73	M	GC	Intestinal	3	N	na	10/03/15	S

The landscape of d16HER2 splice variant expression across HER2-positive cancers

Chiara Costanza Volpi[1], Filippo Pietrantonio[2,3], Annunziata Gloghini[1], Giovanni Fucà[2], Silvia Giordano[4], Simona Corso[4], Giancarlo Pruneri[1], Maria Antista[2], Chiara Cremolini[5], Elena Fasano[6], Serena Saggio[2], Simona Faraci[6], Maria Di Bartolomeo[2], Filippo de Braud[2,3], Massimo Di Nicola[2], Elda Tagliabue[6], Serenella Maria Pupa[6] and Lorenzo Castagnoli[6]

CCV[1] and FP[2,3] contributed equally.

SMP[6] and LC[6] contributed equally.

Supplementary Table S5. Clinical and pathological information of CRC cases.

No.	Age	Sex	Primary tumor location	Mucinous histotype	Grade	Primary tumor resected	pTNM	Date of metastases	synchronous / metachronous	RAS	BRAF
**39**	52	M	left	No	3	Y	pT3 pN1b	01/11/2015	S	WT	WT
**40**	75	M	rectal	No	2	Y	pT3 pN1c	01/11/2009	S	WT	WT
**41**	55	F	rectal	No	2	Y	pT4 pN1b	25/11/2013	S	WT	WT
**42**	46	M	rectal	No	2	Y	pT3 pN1b	02/07/2013	M	WT	WT
**43**	15	M	right	No	2	Y	pT3 pN2	06/04/2015	S	WT	WT
**44**	65	M	right	No	3	Y	pT3 pN1a	01/03/2015	S	G12D	NA
**45**	65	M	left	No	2	Y	pT4a pN0	24/11/2014	S	WT	WT
**46**	65	M	left	No	3	N	na	27/03/2017	S	WT	WT
**47**	38	F	rectal	No	2	Y	pT3 pN0	18/02/2009	S	WT	WT
**48**	76	F	right	No	3	Y	pT3 pN2	03/02/2014	S	WT	WT
**49**	65	F	left	No	2	Y	pT4a pN2a	20/11/2012	M	WT	WT
**50**	68	M	left	No	2	Y	pT3 pN0	01/06/2015	M	WT	WT
**51**	78	M	right	Yes	3	Y	pT4 pN0	01/12/2014	S	WT	D594G
